# School Nursing in a Pandemic: Striving for Excellence in Santa Fe Public Schools

**DOI:** 10.1177/1942602X211005166

**Published:** 2021-04-13

**Authors:** Myrna Barbee-Lee, Kimber Seymour, Anita L. Hett, Gillian Norris, Shona Stack, Allana Cartier, Patricia Haycox, Leeann Armstrong, Laarni Herbert

**Affiliations:** School Nurse, Piñon Elementary School, Santa Fe, NM; School Nurse, Cesar Chavez Elementary School, Santa Fe, NM; Lead Nurse, Santa Fe Public Schools, Santa Fe, NM; School Nurse, Santa Fe High School, Santa Fe, NM; School Nurse, Atalaya & Carlos Gilbert Elementary Schools, Santa Fe, NM; School Nurse, Aspen Community School, Santa Fe, NM; Medically Fragile Nurse, El Camino Real Academy, Santa Fe, NM; Medically Fragile Nurse, Santa Fe High School, Santa Fe, NM; School Nurse, EJ Martinez Elementary School, Santa Fe, NM

**Keywords:** COVID-19, school nurses, hybrid education, public health

## Abstract

When the COVID-19 (coronavirus disease 2019) pandemic led to school closures around the nation in March 2020, the role of the school nurse changed significantly, and it has continued to evolve as districts grapple with how to safely meet students’ academic needs while also protecting the health of their communities. Nurses working for Santa Fe Public Schools in New Mexico have taken their new roles seriously and have been working closely with their district leaders, the New Mexico Department of Health, School Health Advocates, and the Public Education Department to facilitate evidence-based policies and procedures. Activities have included cohorting, contact tracing, resource development, education (of staff and families), planning and implementation of safety procedures, coordination of surveillance testing, and staff screening, along with finding new, COVID safe ways to provide standard school nursing services, including immunization administration, hearing and vision screening, teaching, and promoting wellness and mental health.

School nurses have historically played an important role in promoting public health within their schools and larger communities. Nurses first began working in schools in New York City in the early 1900s with the goal of controlling the spread of communicable diseases among students (Schumacher, 2002). More recently, Mary Pappas, RN, BSN, a school nurse, was the first to recognize and report an H1N1 outbreak in 2009 (Pappas, 2011). Since the onset of the coronavirus disease 2019 (COVID-19) pandemic, our responsibility to care for students and their families while simultaneously considering the public health implications of our work has never been more important.

Santa Fe Public Schools (SFPS) nursing duties mirror those of our peers across the United States. For example, we are responsible for vaccine compliance, hearing and vision screenings, case management for students with Section 504 Accommodation Plans, and the writing and implementation of Individualized Education Plan health plans. In addition, we care for students with chronic conditions, assist students and staff with acute illnesses and injuries, implement school-based vaccine clinics, teach sex education, and provide staff education. Many of us also provide nursing care to medically fragile students. We work closely with our school wellness teams, local health department, and community partners to meet student and family needs.

When COVID-19 hit, our roles shifted and priorities changed. Our relationship with the New Mexico Department of Health (NMDOH) expanded, and we found new ways to support our students, families, and community, embodying the National Association of School Nurse’s (NASN) Framework for 21st Century School Nursing Practice^®^ (Framework; NASN, 2016). We reviewed literature, advocated for health, implemented policies, and created evidence-based documents to support our procedures. As nurse leaders, our practices evolved to reflect a deeper alignment with the Framework’s key principles: Care Coordination, Leadership, Quality Improvement, Community/Public Health, and Standards of Practice. Our hope is that other school nurses might benefit from our work. When reading about our experiences related to COVID-19, it is important to note that the NMDOH (2020) has stated that “anyone who [has] spent 3 or more cumulative minutes within 6 feet of an infected person is considered a close contact,” regardless of if the individuals are wearing a mask or not (p. 1). The Centers for Disease Control and Prevention (CDC, 2020) and many other state health departments define it as being within 6 feet for 15 minutes or longer, making our designation of close contacts much more conservative. Our state governmental agencies, including NMDOH, Public Education Department (PED), and our leaders, have approached the pandemic with consistent dedication to the safety and health of New Mexicans, enabling school nurses to successfully implement protective measures.

## Safe Provision of Nursing Services

One of the major changes in SFPS school nursing practice has involved finding new, safer ways to provide services. We created and implemented innovations to safely administer vaccines, conduct hearing and vision screenings, teach sexual health education, and support wellness in the remote educational setting.

### Drive-Up Vaccine Clinics

SFPS is a Vaccines for Children (VFC) provider, allowing us to administer vaccines at our district office (and for nonfrozen vaccines, at school sites). Our lead nurse and administrative health assistant have ensured maintenance of the pharmacy license, inventory, cold chain, and supplies required by the VFC; and 93.6% of SFPS students are currently vaccine compliant, largely due to this ability and effort. Achieving vaccine compliance during the pandemic presented new challenges. Preparing for the fall 2020 semester, many parents and guardians, when speaking to school nurses, expressed hesitancy in taking their children to see providers or did not understand why vaccines were still required when the students were not going to be physically present in schools. The SFPS nurses provided education and resources to address both concerns. Weekly drive-up vaccine clinics were initiated at the district office, staffed by volunteer nurses. As of February 2021, participating nurses have given 432 vaccines per this initiative. Individual school nurses coordinated with the district office to schedule their students’ appointments. Nurses staffing the clinics made reminder calls on the day or morning prior to appointments. When families arrived, they called to report they were in the parking lot. Nurses obtained verbal confirmation that all people in the vehicle were masked and free of COVID-19 symptoms. Nurses then donned the personal protective equipment (PPE), met the vehicle, obtained consent forms, took the student’s temperature, and screened to determine they met the criteria for immunization. If so, the nurse returned inside to prepare the necessary vaccines. Vaccines were then administered through an open vehicle window, where students remained seated. Parents or guardians received an updated immunization record and the student’s school nurse was notified of students’ compliance. While the drive-up vaccine clinics focused on vaccines required for school, nurses also facilitated several drive-up influenza vaccine clinics. Our district nurses plan to be involved with the impending roll out of the COVID-19 vaccines once they are available to school staff.

### Hearing, Vision, and Dental Screening

New Mexico’s school nurses complete hearing and vision screenings for all pre-K, kindergarten, first-, and third-grade students along with students receiving special education services or being evaluated for learning difficulties. Additionally, pre-K students have the opportunity for a free dental exam provided by NMDOH’s Office of Oral Health. SFPS partners with NM Lions Operation Kid’s Sight to obtain vouchers for free exams and glasses for students without vision coverage. This year, the majority of students remained in remote learning, disrupting screening opportunities. For this reason, SFPS nurses were tasked with querying parents and guardians regarding any new concerns about their child’s hearing or vision (and dental health for students in pre-K). This questionnaire allowed nurses to generate a list of students requiring in-person screening. Several nurses then developed a plan to safely conduct necessary screenings. They worked with the SFPS Office of Student Wellness (OSW) to secure an empty room at the district office. Individual school nurses and health assistants coordinated with parents, guardians, and the screening nurses to schedule the students. The nurses maintained COVID-19 safety protocols while screening for hearing and vision. They enforced social distancing, mask use, and sanitized surfaces between students. As of February 2021, upward of 70 students were screened. Dental referrals are also provided to those with concerns, in lieu of oral screenings.

### Virtual Sexual Health Education

New Mexico requires sexual health education for students in fourth through ninth grades. In SFPS, school nurses teach and coordinate the lessons. Middle school nurses work with community partners to deliver the curriculum. Many middle school nurses began to teach their lessons during the fall semester. At Aspen Community School, for example, middle school sexual health education week took place during the week of December 3 and included eight asynchronous lessons. The nurse also included a synchronous learning period, providing students an opportunity for questions and discussion. In the spirit of innovation, inspired by a transition to remote and hybrid learning, the anatomy and physiology portion of middle school sexual education, normally taught by the school nurses, was converted to an online platform by a district nurse leader. Members of SFPS’s nurse leadership team (NLT) are currently working on translating our elementary curriculum into virtual presentations, to be taught synchronously to students with parent/guardian’s consent (as it would be during on-campus education).

### Supporting Wellness and Mental Health

The SFPS OSW is composed of school nurses as well as school counselors, prevention and restorative justice programs, support for teen parents, and the Adelante program. The SFPA Adelante (Spanish for “come on in”) provides services for children, teens, and their families experiencing homelessness in accordance with the McKinney-Vento Homeless Assistance Act. During the 2020-2021 school year, the OSW implemented a HIPAA (Health Insurance Portability and Accountability Act)-compliant standardized student wellness check-in form. Completed daily, the form gives students the opportunity to ask for help (academic, medical, emotional, etc.) Often, COVID-19 illness and losses were disclosed. The forms helped identify all manner of student wellness and mental health needs so that district staff, including counselors, social workers, nurses, and administrators can reach out and offer assistance accordingly. They are reviewed daily by the school wellness teams so that student needs can be addressed immediately.

Many schools have also enacted extended learning opportunities for remote students. At Piñon Elementary, for example, several staff members created virtual extracurricular “clubs” to give students an outlet for safe socialization with peers and nonacademic leisure activities. They have prioritized inviting students who seem lonely or isolated. A pioneering school nurse at EJ Martinez Elementary further promoted student wellness by creating a virtual nurse’s office using an online learning platform after which several other nurses have adapted the concept for their own schools. This online space is accessible to all students, staff, and families and includes opportunities to interact with the school nurses. It also serves as a repository for evidence-based health information and a direct link to SFPS health policies and procedures.

## Safe Hybrid Education

SFPS implemented a voluntary (for students and staff) hybrid education program at the elementary level on October 26, 2020. Preparations and protocols were developed based on a comprehensive COVID-19 tool kit issued by the PED, with congruent support and training from the NMDOH. Teachers started with no more than five students in each of their two groups (A and B). Group A attended on Mondays and Tuesdays and Group B on Thursdays and Fridays. Wednesday was a deep cleaning day with all students and staff working remotely. Nursing duties included training staff and educating families on the CDC, NMDOH, PED, and SFPS rules and regulations for hybrid. Many district nurses participated in the virtual “Q and A” sessions with parents to answer questions related to the new procedures and student safety. We assisted with site-specific planning for cohorting and cleaning, enforcing masks and social distancing, identifying and planning for isolation rooms, and coordinating and enforcing daily staff screening and weekly surveillance testing. We enforced school exclusions for students and staff who were symptomatic and/or exposed, and for any who had traveled out of state or country (per state restrictions). When positive cases began to occur on campuses, we handled reporting and contact tracing, worked to provide families with testing information, and educated them on quarantine and isolation requirements.

### Mandatory Masks, PPE, and Social Distancing

**Figure 1.**
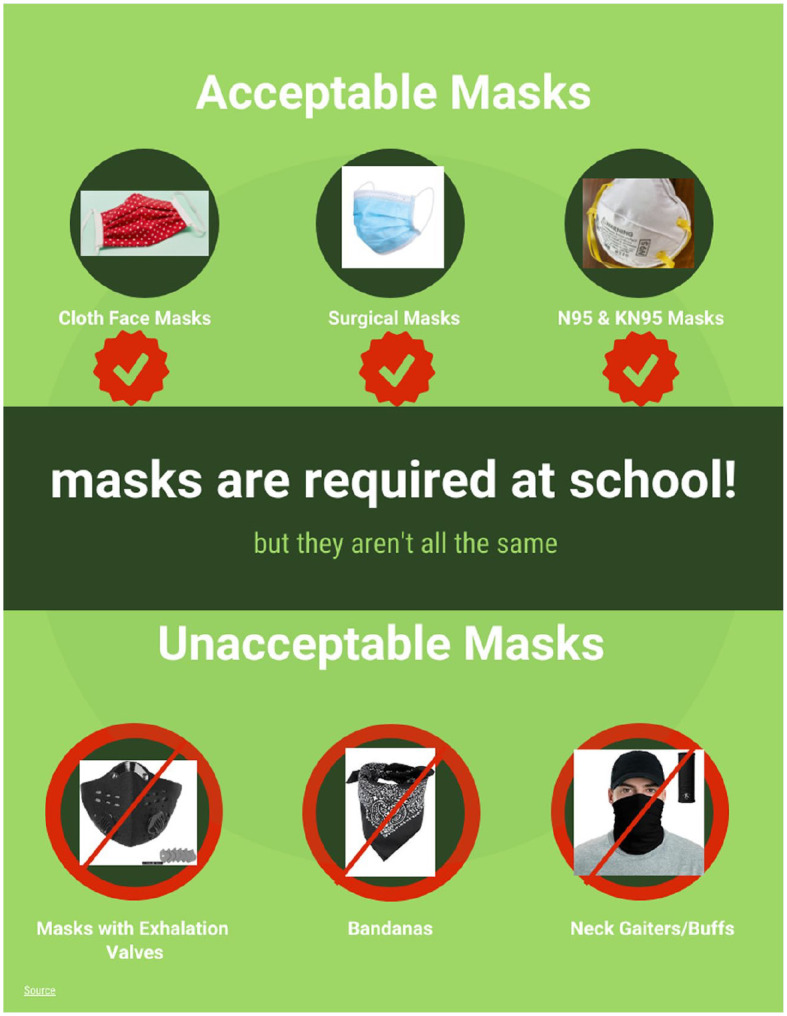
This document illustrates Santa Fe Public Schools mask requirements and was distributed to families prior to their students attending hybrid. It was created by Myrna Barbee-Lee

In accordance with our statewide mandate (established May 2020), masks were required for everyone on campus during hybrid education. The document shown in Figure 1, designed by an SFPS nurse to illustrate SFPS mask requirements, was distributed to families prior to starting hybrid (see online Supplemental files for the Spanish version). SFPS purchased cloth masks and surgical masks for each student and staff member, and PPE (KN95s, N95s, face shields, gloves, and gowns) for staff who required them (i.e., nurses caring for symptomatic students, administering nebulizer treatments, and preschool or special education staff diapering or toileting students). One nurse created a Respiratory Protection Plan per the U.S. Department of Labor (n.d.) and DOH requirements and provided N95 fit testing to staff. SFPS facilities staff installed signage reiterating key messaging and floor labeling to demonstrate appropriate distancing in all schools. Desks were placed 9 feet apart, and staff frequently reminded students to maintain distance while in school.

### Cohorting

Cohorting was essential to preventing virus spread within schools. Although the specifics varied by school, the concept was implemented universally throughout the district. Per PED guidelines, cohorts began with no more than five students, with some expanding later. A cohort received all their educational services together, as well as breakfast, lunch, and recess. This resulted in very few close contacts when tracing positive cases.

### Isolation Rooms

Prior to the start of hybrid education, school nurses were responsible for identifying an isolation room on their campus. Although not hospital-grade isolation rooms, these designated areas housed symptomatic students or staff until they could leave the campus. Requirements included a dedicated bathroom, sink, having student(s) located within the line of sight of a staff member, and ideally located near an exit. Nurses also continue to care for students with chronic conditions and to treat injuries elsewhere, meaning two separate nursing spaces were established at each school.

### Surveillance Testing

Per PED, weekly surveillance testing of a percentage of all on-campus staff is required for districts offering hybrid education. School nurses are responsible for selecting staff, providing sign up information, and following up on test results. In Santa Fe, NMDOH sites provided free testing during the fall semester for the required 5% of staff weekly. PED has increased the spring semester requirement to 12.5% of staff in Santa Fe County, with employees utilizing at-home saliva tests. SFPS nurses continue to lead coordination of surveillance testing.

### Staff and Student Screening

PED also requires daily staff screening (including a temperature check) for anyone who is working at a school site. NLT members worked together to create the *SFPS Daily Screening Tool* form that school staff use to report their self-screening responses in a way that easily allows nurses to review and ensure compliance. SFPS will soon be switching to an app that staff will use to complete their screening. This will hopefully increase staff compliance, especially in departments where staff do not regularly use their district email.

## COVID-19 Tracking/Contact Tracing

**Figure 2.**
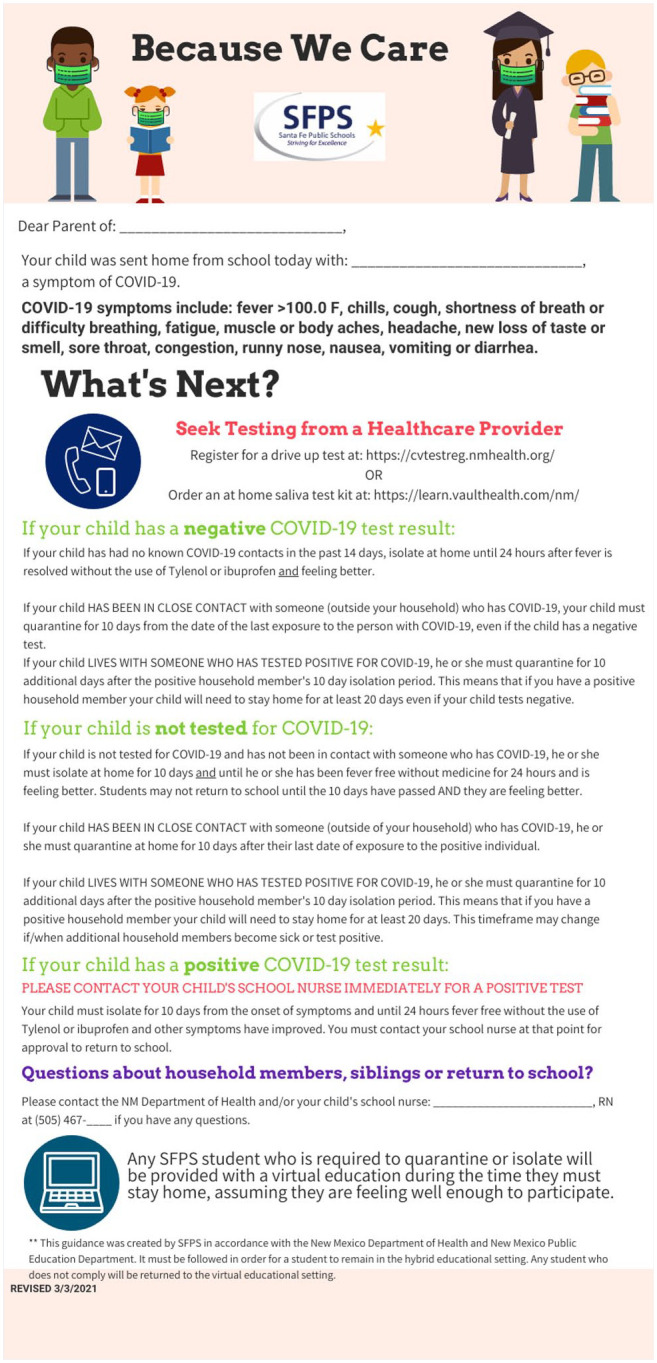
This document was created by an SFPS nurse to provide clear instruction to families of symptomatic students being sent home from school *Note*. SFPS = Santa Fe Public Schools; COVID-19 = coronavirus disease 2019.

School nurses within SFPS were charged with tracking symptomatic students and staff, positives, and close contacts within our individual school communities and reporting positive cases to NMDOH, contact tracing within our school communities (including notification of close contacts), and notifying the state environment department when indicated requesting deep cleaning of affected areas. A standardized script for calling positives and close contacts is provided online in a Supplemental file. During hybrid education, we managed symptomatic students and staff. including making testing referrals and enforcing school exclusions (see Figure 2 for our policies regarding symptomatic students on campus provided in a pamphlet titled “Because We Care”).

**Figure 3.**
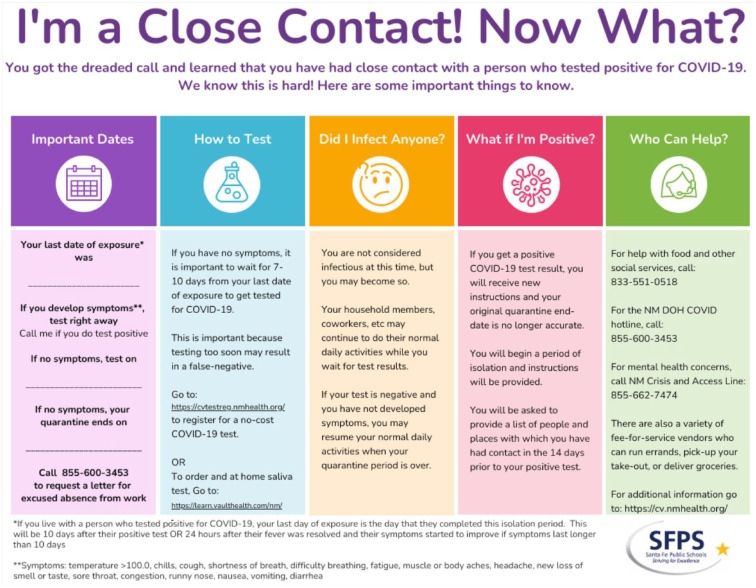
This informational graphic was created by an SFPS nurse Gillian Norris and is used by district nurses to guide contact tracing *Note*. SFPS = Santa Fe Public Schools; COVID = coronavirus disease 2019; NMDOH = New Mexico Department of Health.

We often found that we were the first healthcare workers to make contact with and to provide comfort and education to individuals who received positive test results via email or text. One of our NLT members, with a prior degree in graphic design, created infographics so successful at consolidating key guidelines and quarantine/isolation calculations for close contacts, positives, and continuous contacts that they have since been adopted for use by the NMDOH (see online Supplemental Figures, as well as Figure 3 “I’m a Close Contact! Now What?”). These infographics have been instrumental in our ability to deliver clear, consistent education, enhancing our conversations with affected individuals. SFPS’s lead nurse fields calls from other district departments and schools with nurse vacancies. The work to complete these duties and corresponding communication with supervisors, human resources, and district administrators is ongoing 7 days a week. The OSW recently approved delegating weekend “COVID Call” to the NLT to relieve the lead nurse of documenting and tracing staff positives reported on weekends. We are honored to be able to provide this service to our coworkers and understand the important public health implications of managing the spread of the virus among our staff and, in turn, our community.

## Lessons Learned

The COVID-19 pandemic is far from over, and while we, in our ever expanding school nursing roles, have persevered through many adaptations to mitigate this public health crisis within our community, we will continue to “strive for excellence” (our district’s motto) until the job is done. We recognize that the situation surrounding the COVID-19 pandemic is constantly changing and that our job descriptions will continue to evolve until in-person education is once again the norm. We hope that sharing our procedures and experiences will benefit our counterparts across the nation, making us nimble and be better prepared to meet public health challenges.

## Supplemental Material

sj-docx-5-nas-10.1177_1942602X211005166 – Supplemental material for School Nursing in a Pandemic: Striving for Excellence in Santa Fe Public Schools Click here for additional data file. Supplemental material, sj-docx-5-nas-10.1177_1942602X211005166 for School Nursing in a Pandemic: Striving for Excellence in Santa Fe Public Schools by Myrna Barbee-Lee, Kimber Seymour, Anita L. Hett, Gillian Norris, Shona Stack, Allana Cartier, Patricia Haycox, Leeann Armstrong and Laarni Herbert in NASN School Nurse

sj-png-1-nas-10.1177_1942602X211005166 – Supplemental material for School Nursing in a Pandemic: Striving for Excellence in Santa Fe Public Schools Click here for additional data file. Supplemental material, sj-png-1-nas-10.1177_1942602X211005166 for School Nursing in a Pandemic: Striving for Excellence in Santa Fe Public Schools by Myrna Barbee-Lee, Kimber Seymour, Anita L. Hett, Gillian Norris, Shona Stack, Allana Cartier, Patricia Haycox, Leeann Armstrong and Laarni Herbert in NASN School Nurse

sj-png-2-nas-10.1177_1942602X211005166 – Supplemental material for School Nursing in a Pandemic: Striving for Excellence in Santa Fe Public Schools Click here for additional data file. Supplemental material, sj-png-2-nas-10.1177_1942602X211005166 for School Nursing in a Pandemic: Striving for Excellence in Santa Fe Public Schools by Myrna Barbee-Lee, Kimber Seymour, Anita L. Hett, Gillian Norris, Shona Stack, Allana Cartier, Patricia Haycox, Leeann Armstrong and Laarni Herbert in NASN School Nurse

sj-png-3-nas-10.1177_1942602X211005166 – Supplemental material for School Nursing in a Pandemic: Striving for Excellence in Santa Fe Public Schools Click here for additional data file. Supplemental material, sj-png-3-nas-10.1177_1942602X211005166 for School Nursing in a Pandemic: Striving for Excellence in Santa Fe Public Schools by Myrna Barbee-Lee, Kimber Seymour, Anita L. Hett, Gillian Norris, Shona Stack, Allana Cartier, Patricia Haycox, Leeann Armstrong and Laarni Herbert in NASN School Nurse

sj-png-4-nas-10.1177_1942602X211005166 – Supplemental material for School Nursing in a Pandemic: Striving for Excellence in Santa Fe Public Schools Click here for additional data file. Supplemental material, sj-png-4-nas-10.1177_1942602X211005166 for School Nursing in a Pandemic: Striving for Excellence in Santa Fe Public Schools by Myrna Barbee-Lee, Kimber Seymour, Anita L. Hett, Gillian Norris, Shona Stack, Allana Cartier, Patricia Haycox, Leeann Armstrong and Laarni Herbert in NASN School Nurse
