# Supplementary material for: School Nursing in a Pandemic: Striving for Excellence in Santa Fe Public Schools
Source: NASN Sch Nurse. 2021 Apr 13;36(5):276–83. doi: 10.1177/1942602X211005166 (PMC8047512; doi:10.1177/1942602X211005166)
Supplement: sj-docx-5-nas-10.1177_1942602X211005166 – Supplemental material for School Nursing in a Pandemic: Striving for Excellence in Santa Fe Public Schools [file sj-docx-5-nas-10.1177_1942602X211005166.docx]

**Script for talking to COVID-19 positive Staff members, Contractors and Parents of Students that test positive for COVID-19 (make a copy, enter info on it, share/send to Anita when complete)**

Good morning/afternoon/evening. My name is__________________. I am the school nurse at _______________. I am contacting you today because I was notified by your supervisor that you have tested positive for COVID-19.

I want to let you know I do have to report this to the Public Education Department (PED) (also New Mexico Environmental Department (NMENV) if an employee or contractor). Your personal health information will remain confidential. I have several questions, but first How are you feeling?

If symptomatic, what date did your symptoms start?

What are/were your symptoms? (*when discussing sx, you can offer general nursing guidance/ comfort measures. And be sure to review sx to seek emergency care for, i.e. sob, chest pain, s’n’s of blood clots):

What date did you begin quarantine?

What date did you go for testing?

What date did you get your test result?

When was the last time you were at work?

If you were at work while contagious (2 days before onset of symptoms or day of positive test with no symptoms), did you come into close contact (closer than 6 feet for 3 minutes or longer) with any other employee, contractor or student?

If so, What are the names and phone numbers of the individuals affiliated with SFPS with whom you were in close contact?

What is your date of birth, mailing address, email and alternate phone number?

It is important for you to continue to self isolate (away from family members) until 10 days after symptom onset (or the date of your test if asymptomatic). That date is ______. The date is the very earliest you could end isolation and be able to return to work, but it is very important that you understand the date is only part of it: your *symptoms* must have marked improvement and you must be fever free without the use of fever-reducing medication for a solid 24 hours. The way many people experience this illness there may be a few days where you feel better but then feel worse again. Not all people are feeling their symptoms improved and are free of fever/ done taking ibuprofen/ Tylenol when the 10 day date comes. If that is the case for you, you go by your illness, not the date: you remain in isolation and do NOT go to work until you feel better, and have a 24 hr period with no fever, no ibuprofen and/for Tylenol. At that point you will also need to follow up with a phone call to SFPS Lead Nurse Anita Hett (204-1975) and/ or your supervisor to be cleared to return to work.

It is also important for any household members to quarantine for 14 days from the last exposure. Please contact any close contacts you may have had outside of work to let them know they should quarantine for 14 days from the date of the last exposure. It is recommended all close contacts get tested for COVID-19 7-10 days post exposure.

| Name of Close Contact at SFPS | Phone number | Notes re: conversation |
| --- | --- | --- |
|  |  |  |
|  |  |  |
|  |  |  |
|  |  |  |
|  |  |  |

This document (Figure 2) contains the standardized language utilized by SFPS nurses when contact tracing within our district. It was created by our Lead Nurse, Anita L. Hett, MSN, RN. Link text : Figure 2
