# Supplementary figures and images for: School Nursing in a Pandemic: Striving for Excellence in Santa Fe Public Schools
Source: NASN Sch Nurse. 2021 Apr 13;36(5):276–83. doi: 10.1177/1942602X211005166 (PMC8047512; doi:10.1177/1942602X211005166)

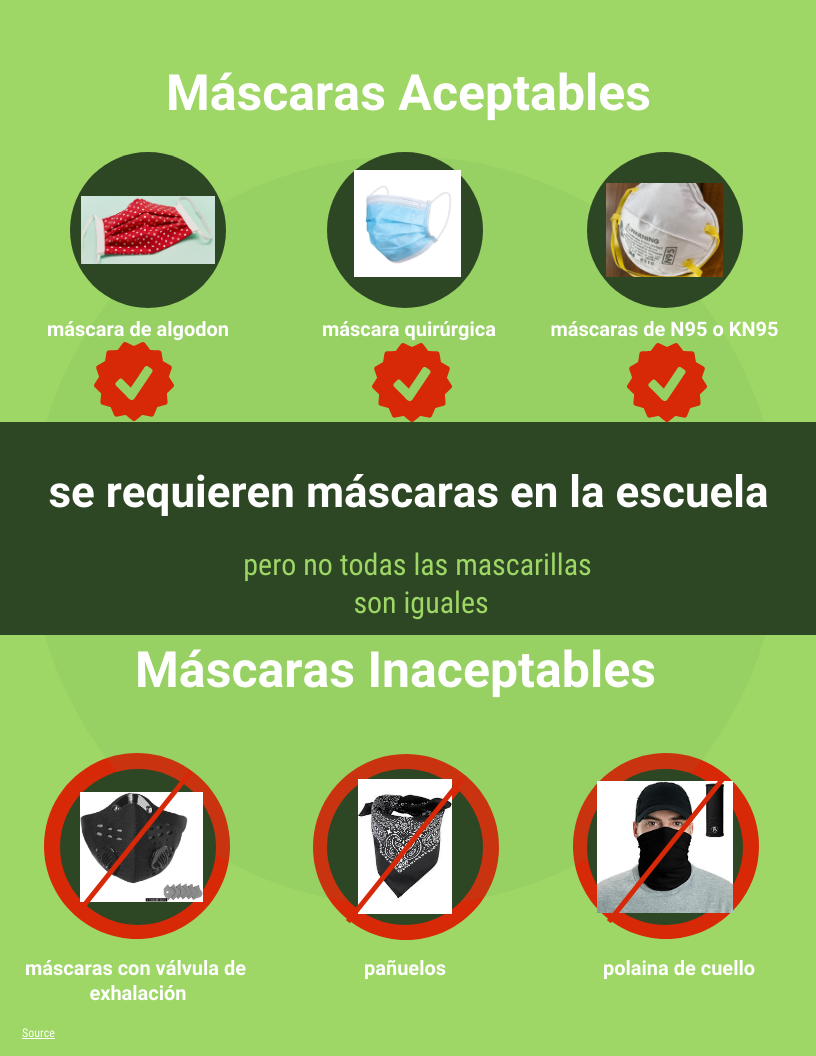

Supplement: sj-png-1-nas-10.1177_1942602X211005166 – Supplemental material for School Nursing in a Pandemic: Striving for Excellence in Santa Fe Public Schools [file sj-png-1-nas-10.1177_1942602X211005166.png]

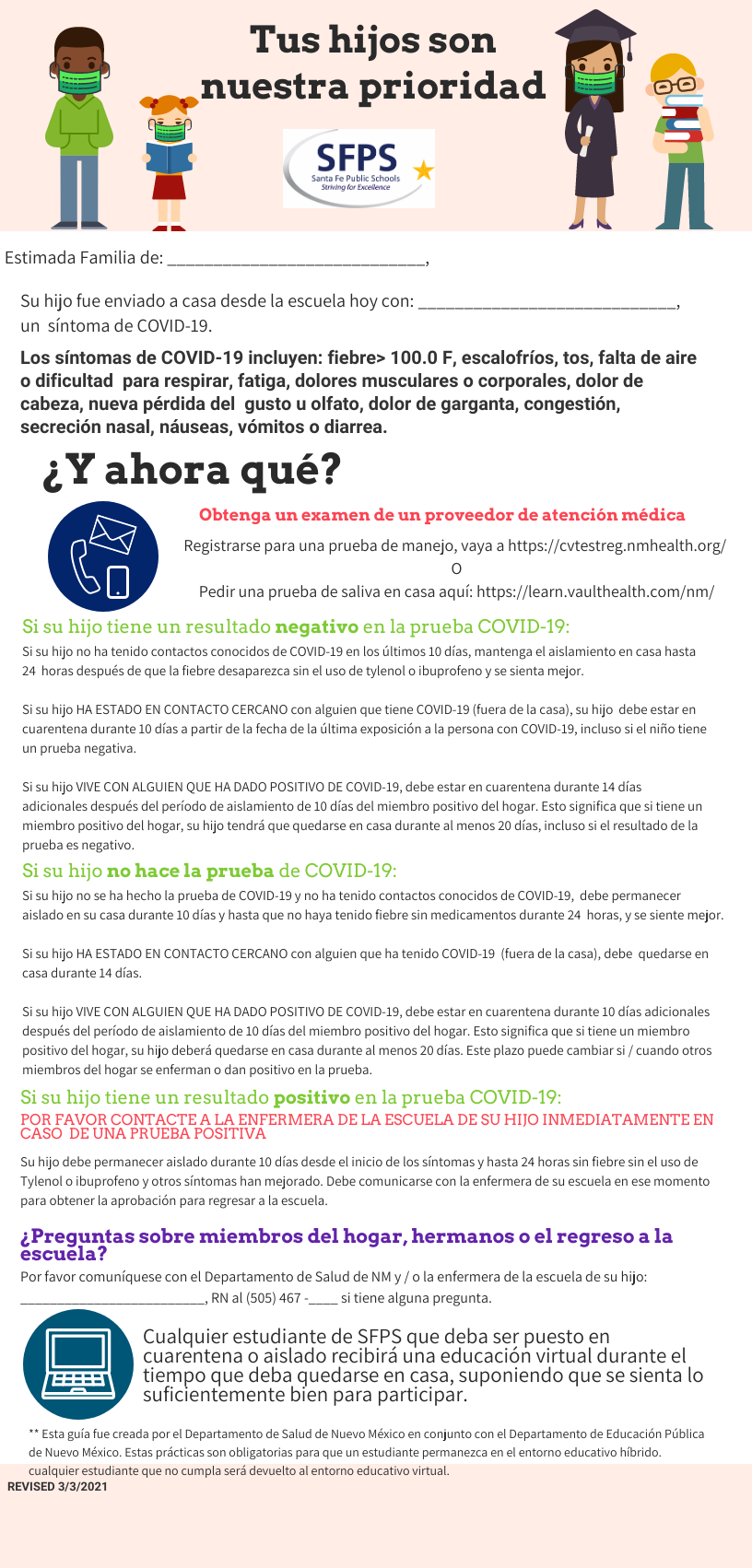

Supplement: sj-png-2-nas-10.1177_1942602X211005166 – Supplemental material for School Nursing in a Pandemic: Striving for Excellence in Santa Fe Public Schools [file sj-png-2-nas-10.1177_1942602X211005166.png]

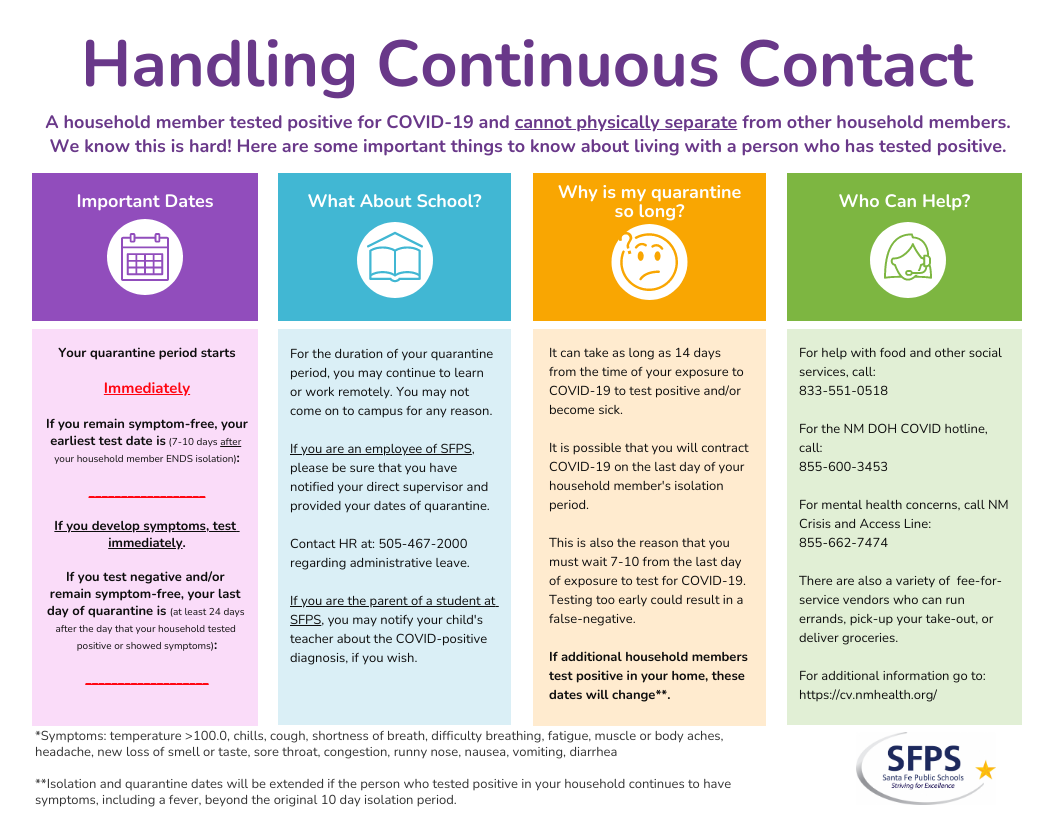

Supplement: sj-png-3-nas-10.1177_1942602X211005166 – Supplemental material for School Nursing in a Pandemic: Striving for Excellence in Santa Fe Public Schools [file sj-png-3-nas-10.1177_1942602X211005166.png]

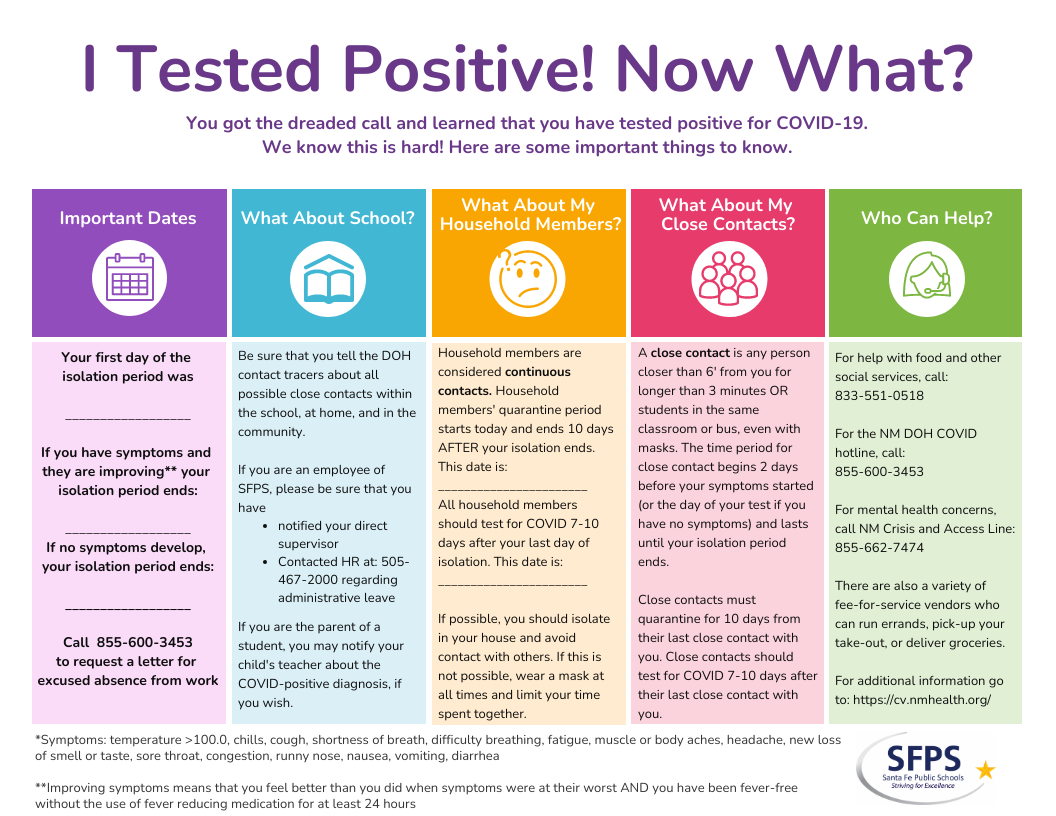

Supplement: sj-png-4-nas-10.1177_1942602X211005166 – Supplemental material for School Nursing in a Pandemic: Striving for Excellence in Santa Fe Public Schools [file sj-png-4-nas-10.1177_1942602X211005166.png]
